# Risk Factors for Tail Alterations on German Dairy Farms

**DOI:** 10.3390/ani16101436

**Published:** 2026-05-08

**Authors:** Rieke Claussen, Roswitha Merle, Marina Volland, Andreas Forkmann, Kerstin-Elisabeth Müller

**Affiliations:** 1 Unit for Internal Medicine and Surgery, Farm Animal Clinic, Division for Ruminants and Camelids, School of Veterinary Medicine, Freie Universität Berlin, 14163 Berlin, Germany; 2 Institute of Veterinary Epidemiology and Biostatistics, School of Veterinary Medicine, Freie Universität Berlin, 14163 Berlin, Germany

**Keywords:** cattle, dairy cow, animal welfare, risk factors, deviated tail, shortened tail

## Abstract

Tail alterations represent an animal welfare concern in dairy cows. The objective of the present study was to identify the risk factors associated with the presence of deviated and shortened tails on 765 German dairy farms. Farm visits, questionnaires, and analyses of milk yield records were conducted, and regression analyses were performed at the animal- and farm-level. At the farm level, a higher percentage of deviated tails in farms was associated with deep cubicles, a higher average oblique rump length of the five largest cows in tie stalls, a higher care ratio, and German Holstein as the predominant breed. At the animal level, lameness in loose housing and higher parity were associated with a higher percentage of deviated tails. The presence of shortened tails was associated with the percentage of deviated tails, as well as with the presence of manure scrapers in loose housing at the farm level, as well as with the higher fat-to-protein ratio in milk at the animal level. The occurrence of tail deviations was associated with health and management factors, while shortened tails were associated with acute injuries or metabolic disorders. Performing longitudinal studies would improve the understanding of the pathogenesis of tail alterations.

## Simple Summary

Tail alterations in dairy cows represent an animal welfare concern. The present study investigated the associated risk factors of deviated and shortened tails on 765 German dairy farms, including 86,355 dairy cows. Regression analyses were performed at the farm and animal level. The following factors were identified as possible contributing factors for deviated tails: lameness, parity, breed, care ratio, deep cubicles in loose housing, and average rump length of the five largest cows in tie stalls. The presence of shortened tails was associated with the presence of deviated tails, the utilization of manure scrapers in loose housing at the farm level, and a higher fat/protein ratio of milk recordings at the animal level. The findings of this study indicate that tail alterations in dairy cows represent a multifactorial condition arising from the interaction of individual animal characteristics and farm management practices. Further research is needed to improve the understanding of the underlying pathogenesis of deviated and shortened tails.

## 1. Introduction

In the bovine species, it is an essential component for the expression of species-specific behavioral patterns; furthermore, the tail serves to chase away insects and to communicate with conspecifics [1,2,3,4]. A number of disorders of the tail in cattle were reported in the past, among these, congenital malformations, inflammatory diseases, and traumatic injuries [5,6,7,8,9]. The latter include lacerations of the skin, phlegmons, and tail tip necroses as well as fractures, subluxations, and luxation of vertebrae causing deviations of the tail axis [7,8,10]. In beef cattle kept indoors on slatted floors or in confined areas in a feedlot, tail injuries are frequently observed. These are related either to direct trauma or—in the case of tail tip necrosis—to feeding rations with a high percentage of starch that cause subclinical rumen acidosis [7,11]. Tail docking (amputation) resulting in shortened tails is indicated for medical reasons in case of injury, necrosis or neoplasms of the tail [12].

Tail docking as a routine procedure in healthy cattle was introduced in New Zealand in the early 1900s to prevent the transmission of leptospirosis to the milking personnel [12]. In addition, farmers argued that tail docking kept the udder clean, thereby reducing the incidence of mastitis. An evaluation of the scientific justification for tail docking came to the conclusion that the intervention is detrimental to the welfare and comfort of the cow [13,14]. For this reason, preventive tail docking is forbidden in most countries, among these the United Kingdom, New Zealand, Ireland, and the USA. The German Animal Welfare Act also generally prohibits amputations of body parts in animals, unless these are performed by a veterinarian for medical reasons [15]. Very recently, alterations of the tail in dairy cows aroused societal interest due to the pain associated with tail injuries in the acute stage and dysfunction of the tail in the chronic stage, both representing serious threats to animal welfare [16,17].

Standard procedures for scoring the tail (basis, tail tip) by visual observation and palpation were developed by scientists from New Zealand and Germany in order to harmonize the tail scoring procedure among assessors from different parts of the world and to define benchmarks for distinct lesions [18,19]. In New Zealand, the prevalence of tail deviations on 200 randomly selected dairy farms was 9.5% (range 0.9–40.3%), and for shortened tails it was 4.5% (range 1.3–10.8%) [19]. In 2–3% of 82 dairy farms in Ireland, the dairy cows examined had tail lacerations, 9% had broken tails, and 8% had docked tails [20]. Data from 765 German dairy farms show that on 25% of the farms, the prevalence of tail alterations was less than 5%, while on another 25% of farms, the herd prevalence exceeded 15%, reaching a maximum of 52.4% [21]. The exact etiology and pathogenesis, however, of the different tail alterations are not fully understood yet [22,23,24].

A project entitled “Animal health, hygiene and biosecurity in German dairy cow operations—a prevalence study (PraeRi)” initiated by the German Federal Ministry of Food and Agriculture (BMEL) and funded by the Federal Agency for Agriculture and Food (BLE) included 765 dairy farms in different regions of Germany [25]. In order to get closer insights into the pathogenesis of tail deviations and shortened tails in dairy cows, the aim of the present study was to evaluate selected risk factors within the framework of the PraeRi project.

## 2. Materials and Methods

### 2.1. General Information

This study took place within the framework of the PraeRi project. It was conducted by three different teams of veterinarians affiliated with the “University of Veterinary Medicine Hannover”, the “Freie Universität Berlin”, and the “Ludwig Maximilians University of Munich”, and 765 dairy farms in three different regions (South, North, East) throughout Germany were included and visited on a single occasion. The sample size per region was calculated for the total study, on different animal health conditions (e.g., lameness and mastitis), assuming a prevalence of 40%, a 95% confidence level, 80% power, and an accuracy of ±5% [26]. It did not specifically consider outcomes related to deviated and shortened tails. The selection of farms was conducted on the basis of data obtained from the National Livestock Information Database (HIT, München, Germany) for the northern and eastern parts of Germany, and from the Milchprüfring Bayern e.V. (Wolnzach, Germany) for the southern region, reflecting a representative sample for the respective region [25,26]. In the North region (Schleswig-Holstein, Lower Saxony), 253 farms were visited; in the East region (Mecklenburg-Western Pomerania, Brandenburg, Thuringia, Saxony-Anhalt), 252; and in the South region (Bavaria), 260. The farmers willingly participated on a voluntary basis and signed a written consent form.

### 2.2. Farm Visit Procedure and Data Collection

The study was designed as a large-scale cross-sectional observational trial, including 765 farms visited on a single occasion between December 2016 and August 2019. A team of 21 veterinarians affiliated with the veterinary universities given above collected data on the farms. Before commencing farm visits, the observers were fully trained in all measures to be assessed by using photographs, videos, and on-farm training. The training included recognition of conditions that could affect animal welfare as well as tail alterations. Subsequently, acceptable intra- and inter-observer reliabilities were achieved.

During the farm visit, a questionnaire was completed by interviews with the dairy farmer or herd manager on general farm characteristics, year of last modernization, daily routine procedures, and farm infrastructure (main occupation or sideline job, organic or conventional farming). The farming and housing systems were evaluated for cubicle design (deep-bedded, mixed, and raised cubicles) and dimensions (cubicle length, width, and height of the neck rail) separately for loose housing and tie stalls. Also, flooring type (predominantly solid/concrete floors; predominantly slatted floors, various types of flooring), access to pasture separated in pasture access for different groups (no pasture access; only for lactating; only for dry cows; for all cows) or per time in tie stalls (no pasture access, ≥2 h, >2–4 h, ≥4 h), and manure removal (presence or absence of manure scraper in all or some compartments; or the presence of automatic manure scrapers) were evaluated. All cows or a representative sample of cows, depending on farm size, were assessed on each farm. The individual ear tag number (last five digits) was recorded. Due to the different farm sizes and agricultural structures in the regions [27], the sampling size for the clinical scoring procedures had to be calculated depending on herd size per region. The maximum sampling size per herd and region was determined as follows: 130 cows for the South region and 213 for the North region. For the East region, a maximum sampling size of 166 was determined for farms with herd sizes of 160–292 dairy cows and 292 for farms with herd sizes ≥293 dairy cows. All measures were recorded for the same sample of animals. If cows were kept in separate compartments (functional housing units within the farm (e.g., the calving area)), proportionate sampling was carried out based on group size. [26,27]. Visual lameness scoring was performed by applying the 5-point scale for cows in loose housing [28]. Cows that received lameness scores <3 were finally assigned to the “non-lame” group, and cows receiving scores ≥3 were assigned to the “lame” group [29]. For tie-stall housing, the Stall Lameness Score (SLS) was applied, evaluating weight-bearing and shifting of feet, sparing a foot while standing, unequal weight bearing when stepping from side to side, and standing at the edge of the curb [30]. Cows displaying two or more criteria were recorded as “lame” [31]. Body condition scoring was performed following the procedure described by Edmonson and modified by Metzner et al. (1993) [32,33]. This score was applied regardless of the cattle’s breed and rounded to the nearest 0.25. In light of the observations on different breeds and lactation stages, the Body condition score (BCS) was classified for each animal by breed and lactation stage [26]. The animals were then categorized into underconditioned, normal, or overconditioned groups [30]. The integument was assessed for cleanliness [34] and technopathies [34,35,36]. The body dimensions of the five largest dairy cows in a herd were assessed by determining the average oblique rump length. Data on milk yield, milk content, parity, age, breed, and days in milk were retrieved from the HIT and from the national milk recording associations over the 12 months prior to the farm visit. 621 farmers provided consent for access to milk yield records. Breed was categorized for predominant breed per farm on farm- level in German Holstein, German Simmental, Brown Swiss and other. Parity was used as the average parity on the farm level. The care ratio was an additional measure on the questionnaire conducted in the East region, which relates the number of cows to the number of full-time workers, and the proportion of part-time workers to the baseline value used in this study is 30 dairy cows per full-time working employee. Quality of silage was classified in different categories: 1 = silage of normal quality; 2 = silage of slightly deficient quality; 3 = silage of significantly deficient quality; 4 = silage that is more or less spoiled [37].

### 2.3. Scoring of Tail Alterations

Lactating and dry cows on site on the day of the farm visit were visually evaluated for tail alterations. These were recorded as follows: 1 = no deviated or shortened tail, 2 = deviated tail, and 3 = shortened tail. “Deviated tails” were defined as tails with a significant axis deviation and/or swelling of the tail. The tail of any cow that could not be traced in its full length was classified as a “shortened tail”. However, a trimmed tail switch was not classified as such. The classification is similar to previous studies [20]. The tail tips were not considered in the present study.

### 2.4. Data Handling

A list including potential welfare-associated risk factors was compiled based on the literature research and expert knowledge. All selected risk factors and confounding variables are listed in Table 1, Table 2, Table 3 and Table 4. For each variable, descriptive analyses were performed at the farm level for each animal using SAS 9.4 (SAS Institute, Cary, NC, USA).

For the multivariable analyses, hypothesis-based approaches were used, with the binary target variable “deviated tail” or “shortened tail”, and all potential influencing variables were identified. For each combination of target and influence factor, a causal directed acyclic graph (DAG) was drawn using DAGitty web application (Institute for Computing and Information Sciences, Radboud University and Medical BioSciences Department, Radboudumc, Nijmegen, The Netherlands) [38]. DAGs were developed separately for loose housing and tie stalls. Variables that had a direct or an indirect link to the influence variable, as well as to the target variable, were defined as confounding variables and included in the multivariable model. Herd size, farming type (organic or conventional), and region were considered general confounders and therefore included in each model.

An univariable model was analyzed for each influence variable, followed by a multivariable model that included the influence variable and all respective confounders. If the potential influence factor was at the animal level, a mixed logistic regression model was used, including farm as a random factor. Only farms with more than six cows affected by deviated or shortened tails were included in the analysis. A total of 375 farms with 66,930 cows had more than six cows with deviated tails, and 38 farms with 7754 cows had more than six cows with shortened tails. If the risk factor was at the farm level, a generalized linear regression model with a negative binomial distribution was used. Odds ratios (ORs, logistic regression) or Incidence Rate Ratios (IRRs, negative binomial regression), including 95% confidence intervals (95% CIs), were calculated for the respective influence factor. *p*-values < 0.05 were considered statistically significant. OR and IRR were obtained by exponentiating the regression coefficients. All estimates were used at full precision and rounded to two decimal places for presentation.

## 3. Results

### 3.1. Deviated Tails

The herd prevalences for deviated tails on German dairy farms ranged from 0% to 52.35% per farm, with a mean of 10.00% [21]. A total of 24 farm-level and 6 animal-level models were analyzed to investigate risk factors associated with deviated tails (Table 1 and Table 2). Eight out of twenty-four influence factors were significant at the univariable farm level. In the adjusted model, only two of the eight models were significant (Table 1). Furthermore, two risk factors that were not significant in the univariable model have become significant in the adjusted model. At the animal level, only two of six models achieved statistical significance (Table 2).

#### 3.1.1. Deviated Tails—Farm Level

In tie stalls, a greater rump length was identified as a risk factor for deviated tails. Larger body dimensions increased the incidence ratio of deviated tails in the adjusted model (*p* = 0.040) on the farm. The IRR of 1.03 indicates that with an increase of 10 cm in rump length, the incidence ratio of deviated tails rose by 30% from the farm’s baseline value. This association was not observed for loose housing (95%CI = 1.00–1.09).

The presence of deep-bedded cubicles in loose housing resulted in an increase in the incidence ratio of deviated tails (*p* = 0.028); on farms exclusively equipped with deep cubicles, a higher percentage of cows with deviated tails was observed (IRR = 1.11; 95%CI = 0.98–1.25).

The care ratio was associated with deviated tails. The higher the care ratio, the higher the probability of deviated tails (*p* = 0.034, IRR = 1.05; 95%CI = 1.00–1.10). Each additional 10 dairy cows per employee increased the baseline value of the incidence ratio by 5%.

Parity was a significant risk factor in the univariable model (*p* ≤ 0.001). This association was no longer significant after adjusting for other variables (*p* = 0.097). The risk increased with higher parity by an IRR of 1.14 (95%CI = 0.98–1.33).

The predominant breed was shown to be an influential factor of relevance in the adjusted model (*p* ≤ 0.001). All breeds, compared with German Holsteins, had a decreased risk of tail deviation. The Simmental breed had the lowest risk (IRR = 0.46, 95% CI = 0.32–0.66) for deviated tails and was highly associated with the outcome (*p* < 0.001).

**Table 1:** Farm-level risk factors for deviated tails on 765 farms in Germany. Results of the univariable analyses and the confounder-adjusted models in loose-housing systems and tie stalls.

	Univariable Analysis				Confounder-Adjusted Model				
Variable	N	Crude Estimate	IRR	*p*-Value	Adjusted Estimate	IRR	95%CI		*p*-Value
**Lameness ^1,2^**	764	0.01	1.01	**<0.001**	0.00	1.00	0.99	1.00	**0.414**
**Who performs hoof trimming ^1^**	761			**<0.001**					**0.774**
Hoof trimmer	566	0.40	1.49	<0.001	0.02	1.30	0.88	1.20	0.774
Farmer	195	.	.	.	.	.	.	.	.
**Width of the cubicle (cm) ^1,3^**	628	−0.03	0.98	**0.01**	0.00	1.00	0.99	1.01	**0.594**
**Length of the cubicle (cm) ^1,4^**	629	0.00	1.00	**0.162**	0.00	1.00	1.00	1.01	**0.261**
**Intermediate width of walking alleys (cm) ^1,5^**	547			**0.146**					**0.226**
<120 cm	116	−0.12	0.88	0.278	−0.13	0.88	0.73	1.06	0.183
120–250 cm	401	0.05	1.05	0.619	−0.01	0.99	0.86	1.16	0.947
>250 cm	90	.	.	.	.	.	.	.	.
**Proportion of neck rails with height > 165 cm ^1,4^**	629			**0.361**					**0.843**
>80%	150	.	.	.	.	.	.	.	.
20–80%	245	0.04	1.04	0.583	0.02	1.02	0.89	1.17	0.773
<20%	234	−0.06	0.94	0.483	−0.02	0.98	0.86	1.13	0.820
**Width of stall platform (tie stalls) (cm) ^1,4^**	92	−0.02	0.98	**0.315**	−0.03	0.97	0.93	1.02	**0.126**
**Length of stall platform (tie stall) (cm) ^1^**	92			**0.969**					**0.809**
170–190 cm	23	0.04	1.04	0.934	0.33	1.39	1.39	4.16	0.555
<170 cm	53	0.09	1.10	0.826	0.33	1.38	0.49	3.89	0.535
≥190 cm	16	.	.	.	.	.	.	.	.
**Average oblique rump length (tie stalls) (cm) ^1^**	89	0.05	1.05	**0.040**	0.03	1.03	1.00	1.09	**0.040**
**Average oblique rump length (in loose-housing systems) (cm)**	389	−0.01	0.99	**0.320**	−0.01	0.99	0.98	1.01	**0.320**
**Deep bedded cubicles ^11^**	637			**0.841**					**0.028**
Consistently deep bedded cubicles	192	0.04	1.04	0.622	0.10	1.11	0.98	1.25	0.098
Mixed	123	−0.01	0.99	0.889	−0.10	0.90	0.79	1.04	0.162
Consistently raised cubicles	322	.	.	.					
**Manure scraper in loose housing ^1,6^**	453			**0.016**					**0.659**
Present in all compartments	106	0.24	1.27	0.082	−0.10	0.91	0.74	1.12	0.368
Present in some compartments	83	0.17	1.18	0.007	−0.04	0.96	0.80	1.15	0.667
None	264	.	.	.	.	.	.	.	.
**Manure scraper in tie stalls ^1,4^**	94			**0.953**					**0.850**
Present	62	0.02	1.02	0.953	−0.06	0.95	0.53	1.70	0.850
Absent	32	.	.	.	.	.	.	.	.
**Robotic manure scraper ^1,7^**				**0.606**					0.728
Present	542	0.05	1.05		.	.	.	.	.
Absent	89	.	.	.	0.03	1.03	0.87	1.21	
**Manure grid (tie stalls) ^1,4^**	93			**0.701**					**0.506**
Present	63	0.11	1.12	0.701	0.23	1.26	0.63	2.52	0.507
Absent	30	.	.	.	.	.	.	.	.
**Manure gutter (tie stalls) ^1,4^**	39			**0.963**					**0.561**
Present	27	−0.02	0.98	0.963	0.34	1.41	0.44	4.50	0.561
Absent	12	.	.	.	.	.	.	.	.
**Pasture access (tie stalls) ^1,8^**	83			**0.196**					**0.137**
No access	46	−0.07	0.48	0.798	0.77	2.17	0.72	6.51	0.169
≥2 h	4	0.99	2.69	0.060	1.42	4.15	1.11	15.51	0.034
>2–4 h	1	−0.53	0.59	0.684	−0.71	0.49	0.04	5.88	0.574
≥4 h	32	.	.	.	.	.	.	.	.
**Proportion of cows lying outside cubicle (%) ^1,9^**	521	0.07	1.08	**0.376**	0.02	1.02	0.90	1.18	**0.745**
**Year of last modernization ^1,10^**	185	0.00	1.00	**0.738**	0.00	1.00	0.99	1.01	**0.754**
**Type of milking parlor ^1^**	668			**<0.001**					**0.330**
Auto-tandem	47	−0.14	0.87	0.280	−0.03	0.97	0.78	1.21	0.7802
Rotary	44	0.36	1.44	0.003	−0.20	0.82	0.67	1.00	0.051
Side-by-side	65	0.24	1.27	0.023	−0.07	0.93	0.78	1.11	0.410
Other	190	−0.27	0.76	0.001	−0.10	0.91	0.79	1.05	0.201
Herringbone	322	.	.	.	.	.	.	.	.
**Care ratio (10 cows/employee) ^11^**	196	0.02	1.02	**0.336**	0.05	1.05	1.00	1.10	**0.034**
**Average parity ^1^**	765	−0.32	0.73	**<0.001**	0.13	1.14	0.98	1.33	**0.098**
**Mastitis treatment on farms with automatic milking systems ^1^**	83	0.01	1.01	**0.187**	0.01	1.00	0.75	1.40	**0.479**
**Predominant breed ^1,12^**	765			**<0.001**					**<0.001**
German Simmental	183	1.04	0.26	<0.001	−0.79	0.46	0.32	0.66	<0.001
Brown Swiss	20	1.04	0.35	<0.001	−0.43	0.65	0.35	1.20	0.168
Other	153	0.30	0.74	0.003	−0.10	0.90	0.74	1.10	0.307
German Holstein	409	.	.	.	.	.	.	.	.

N = number of farms, OR = odds ratio, IRR = incidence rate ratio, 95% CI = 95% confidence interval. ^1^ Adjusted for herd size, farming type, breed, region, ^2^ adjusted for average oblique rump length (in cm observed by the 5 largest dairy cows per farm), parity, ^3^ adjusted for average oblique rump length (in cm observed by the 5 largest dairy cows per farm), deep bedding in cubicles, ^4^ adjusted for average oblique rump length (in cm observed by the 5 largest dairy cows per farm), ^5^ adjusted for access to pasture or to a paddock, ^6^ adjusted for flooring type, ^7^ adjusted for herd size, farming type, breed, region, pasture access in some groups, animal-stall ratio (mean), smallest alley width (in cm). Cows lying outside the cubicles, ^8^ adjusted for farm ran part- or full-time, ^9^ adjusted for pasture access, animal/stall ratio, smallest alley width (in cm), robotic manure scraper, ^10^ adjusted for year of construction, ^11^ adjusted for educational background of manager, ^12^ adjusted for parlor type, automated milking system.

#### 3.1.2. Deviated Tails—Animal Level

Lameness exerts a marked effect on the prevalence of deviated tails (*p* < 0.001). Lower odds of tail deviations were observed in cows that were not lame (OR = 0.77, 95% CI = 0.70–0.85). A nearly identical effect was also observed for tie-stall housing systems (OR = 0.769, 95% CI = 0.48–1.20). However, the adjusted model was not significant (*p* = 0.284). Parity had a significant effect on the odds of deviated tails for individual dairy cows (*p* < 0.001). The risk of a deviated tail was 47% (OR = 0.53 and 95% CI = 0.46–0.62) lower in the first parity compared to the fifth parity after adjustment. The OR decreased with each subsequent parity, with no further significance detected between the fourth and fifth parities (*p* = 0.236).

**Table 2:** Risk factors for deviated tails—animal level. Results of univariable analyses and adjusted models on 375 dairy farms in three different regions (North, South, East).

	Univariable Analysis				Confounder-Adjusted Model				
Variable	N	Crude Estimate	OR	*p*-Value	Adjusted Estimate	OR	95%CI		*p*-Value
**Lameness in loose housing ^1,2^**	18,290			**<0.001**					**<0.001**
No	11,832	−0.28	0.75	<0.001	−0.27	0.77	0.69	0.85	<0.001
Yes	6458	.	.	.	.	.	.	.	.
**Lameness in tie-stall housing ^1,2^**	2263			**0.393**					**0.2836**
No	1812	−0.24	0.79	0.329	−0.26	0.77	0.48	1.20	0.284
Yes	451	.	.	.	.	.	.	.	.
**Hock lesions in loose housing ^1,3^**	**50,368**			**0.338**					**0.525**
No integument alterations	15,088	.	.	.	.	.	.	.	.
Hairless spot	26,298	0.18	1.20	0.639	−0.04	0.96	0.43	2.17	0.922
Swelling and/or wound	8982	0.64	1.9	0.159	0.41	1.5	0.59	3.8	0.394
**Hock lesions in tie stalls ^1,3^**	1756			**0.794**					**0.765**
No integument alterations	395	.	.	.	.	.	.	.	.
Hairless spot	998	−0.19	0.83	0.504	−0.22	0.52	0.44	1.45	0.470
Swelling and/or wound	363	−0.17	0.84	0.621	−0.21	0.81	0.39	1.70	0.5822
**Body condition score ^1^**	**49,831**			**0.707**					**0.9102**
Underconditioned	11,922	0.31	1.37	0.470	0.09	1.20	0.46	2.64	0.8354
Optimum	18,413	0.32		0.44	0.18	1.20	0.52	2.74	0.6704
Overconditioned	19,496	.	.	.	.	.			
**Parity ^1^**	17,745			**<0.001**					**<0.001**
1st	16,982	−0.64	0.53	<0.001	−0.63	0.53	0.46	0.62	<0.001
2nd	13,017	−0.48	0.62	<0.001	−0.47	0.63	0.54	0.73	<0.001
3rd	8804	−0.30	0.74	<0.001	−0.29	0.75	0.64	0.88	<0.001
4th	5113	−0.11	0.90	0.218	−0.11	0.90	0.76	1.07	0.236
5th and higher	4772	.	.	.	.	.	.	.	.

N = number of animals, OR = odds ratio, 95% CI = 95% confidence interval. ^1^ Adjusted for herd size, farming type, breed, region, average oblique rump length (in cm observed by the 5 largest dairy cows per farm), ^2^ adjusted for parity, ^3^ adjusted for deep cubicles, neck rail position (median in cm).

### 3.2. Shortened Tails

The data from this cross-sectional study revealed herd prevalences for shortened tails ranging from 0% to 20.42%, with a mean of 1.07% [21]. Twenty-five farm-level models (Table 3) and two animal-level models (Table 4) were used to identify the risk factors associated with shortened tails in dairy cows.

#### 3.2.1. Shortened Tails—Farm Level

The incidence ratio for shortened tails was higher in the presence of tail deviations in the adjusted model (IRR = 1.44, *p* < 0.001, 95% CI = 1.22–1.71).

Lameness was neither identified in loose housing (*p* = 0.322, IRR = 0.99, 95%CI = 0.98–1.01) nor in tie-stall systems (*p* = 0.8665, IRR = 1.00, 95%CI = 0.95–1.05) as an associated risk factor for shortened tails after adjustment. In loose-housing systems, the risk of shortened tails was higher when manure scrapers were present in all compartments (IRR = 1.89, 95%CI) or in some compartments (IRR = 1.48). This effect could not be reproduced in tie stalls or with robotic manure scrapers.

Although the effect of deep cubicles was not statistically significant (*p* = 0.081), on farms that exclusively provided deep cubicles, a lower probability of shortened tails was observed compared with farms that kept their cows exclusively on raised cubicles (IRR = 0.70, 95% CI 0.51–0.98).

To investigate the effect of dimensions and hygiene of cubicles and walking alleys, floor contamination, the proportion of cows staying outside the cubicles, moisture on the floor, and soiled distal limbs were evaluated. The percentage of soiled distal limbs turned out to have a slight but non-significant positive effect on the risk of shortened tails.

Across the different housing systems, straw-based housing had a lower risk ratio than tie-stall systems. The higher risk ratio was most pronounced for pasture-based systems (IRR = 1.46) and for those with various housing systems in one lactation (IRR = 1.67).

The BCS (*p* = 0.6254) and the fat/protein ratio in milk (*p* = 0.4146) did not have significant effects on the risk of shortened tails.

**Table 3:** Farm-level risk factors for shortened tails on 765 farms in Germany. Results of the univariable analyses and the confounder-adjusted models in loose-housing systems and tie stalls.

	Univariable Analysis				Confounder-Adjusted Model				
Variable	N	Crude Estimate	OR	*p*-Value	Adjusted Estimate	IRR	95%CI		*p*-Value
**Deviated tails (log.) ^1,2^**	693	0.24	1.27	**0.001**	0.36	1.44	1.22	1.71	**<0.001**
**Lameness in loose-housing system ^1,3^**	456	−0.01	0.9	**0.228**	−0.01	0.99	0.98	1.01	**0.322**
**Lameness in tie-stall housing system ^1,3^**	80	−0.01	0.99	**0.676**	0.00	1.00	0.95	1.05	0.867
**Manure scraper in loose housing ^1,4^**				**0.489**					**0.021**
In all compartments	106	0.21	1.24	0.656	0.64	1.89	0.99	2.20	0.012
In some compartments	83	0.08	1.08	0.236	0.39	1.48	1.15	3.11	0.054
No	264	.	.	.	.	.	.	.	.
**Manure scraper in tie stalls ^1^**	80			0.470					0.301
In at least one compartment	6	−0.50	0,61	0.486	−0.80	0.45	0.10	2.02	0.301
No	1	.	.	**.**	.	.	.	.	**.**
**Robotic manure scraper ^1,5^**	452	−0.34	0.71	**0.062**	−0.15	0.86	0.59	1.26	**0.447**
**Deep bedded cubicles ^1,^**				**0.089**					**0.081**
Consistently deep cubicles	132	−0.30	0.74	0.083	−0.35	0.70	0.51	0.98	0.038
Mixed	113	0.11	1.11	0.522	0.00	1.00	0.73	1.38	0.998
Consistently raised cubicles	211								
**Length of stall platform (tie stall) ^1,4^**	76			0.342					0.257
<170 cm	43	−0.70	0.43	0.368	−1.27	0.28	0.05	1.57	0.149
≥170 to <190 cm	20	−0.35	0.71	0.684	−0.25	0.78	0.14	43.7	0.779
≥190 cm	13	.	.	.	.	.	.	.	.
**Floor contamination ^1,7^**	454			0.459					**0.632**
Low to medium soiling (<50% of the surface)	222	.	.	.	.	.	.	.	.
Severe soiling (>50% of the surface)	130	−0.18	0.83	0.267	−0.16	0.85	0.62	1.17	0.316
Completely covered with feces	14	−0.61	0.54	0.194	−0.40	0.67	0.27	1.65	0.381
Clean or single cow paths	88	−0.06	0.95	0.756	0.00	1.00	0.61	1.42	0.992
**Proportion of cows lying outside cubicle (%) ^1,7^**	402	−0.04	0.96	**0.100**	−0.03	0.97	0.93	1.02	**0.211**
**Soiled lower legs (%) ^1,8^**	452	0.01	1.01	**0.007**	0.01	1.01	1.00	1.01	**0.158**
**Soil moisture ^1,6^**				**0.857**					**0.313**
Ponding > 50% of surface	23	.	.	.	.	.	.	.	.
No ponding	53	0.02	1.02	0.948	−0.12	0.89	0.44	1.77	0.731
Sporadic ponding	201	0.12	1.13	0.700	0.20	1.22	0.66	2.24	0.524
**Average oblique rump length (cm) (loose-housing system) ^1^**	389	0.026	1.07	**0.017**	−0.01	0.99	0.97	1.02	**0.55**
**Predominant housing system ^1^**	681			**0.040**					**0.591**
Loose housing with cubicles	456	0.50	1.66	0.099	0.10	1.11	0.58	2.13	0.759
Straw-based	108	0.27	1.32	0.412	−0.08	0.92	0.47	1.87	0.823
Various	5	0.64	1.89	0.479	0.53	1.67	0.29	9.84	0.554
Pasture access	31	1.11	3.03	0.007	0.38	1.46	0.63	3.41	0.376
Tie stall	81	.	.	.	.	.	.	.	.
**Type of milking parlors ^1^**	513			**0.092**					**0.690**
Herringbone	278	.	.	.	.	.	.	.	.
Rotary	44	−0.36	0.70	0.106	−0.02	0.98	0.61	1.57	0.937
Side-by-side	65	−0.10	0.91	0.636	−0.15	0.86	0.59	1,26	0.442
Other	86	−0.32	0.72	0.104	−0.12	0.88	0.60	1.57	0.537
Auto-tandem	40	−0.68	0.51	0.027	−0.39	0.68	0.38	1.22	0.537
**Robotic milking system ^1^**				**0.200**					**0.164**
No	366	0.24	1.27	0.197	0.25	1.28	0.91	1.81	0.161
Yes	88	.	.	.	.	.	.	.	.
**Animal/stall ratio (median) ^1^**	584	0.37	1.45	0.313	0.03	1.03	0.50	2.13	0.929
**Predominant breed ^1^**	765			**0.027**					**0.664**
German Simmental	183	−0.45	0.64	0.009	−0.23	0.32	0.43	1.48	0.47
Brown Swiss	20	−0.89	0.41	0.132	−0.57	0.65	0.16	1.48	0.383
Other	153	−0.09	0.92	0.574	0.07	0.17	0.77	2.02	0.697
German Holstein	409	.	.	.	.	.	.	.	.
**Veterinary herd health management ^1^**	7 63			**0.957**					**0.243**
No	427	−0.01	0.99	0.957	0.14	1.15	0.91	1.46	0.244
Yes	336	.	.	.	.	.	.	.	.
**Care ratio ^1^**	196	0.00	1.00	**0.781**	0.01	1.01	0.99	1.02	**0.254**
**Median ECM ^1,9^**	660	0.00	1.00	**0.843**	−0.02	0.98	0.99	1.02	**0.299**
**Fat/protein ratio in milk ^1,9^**	719	−0.01	0.99	**0.013**	0.00	1.00	0.99	1.00	**0.415**
**Maximum of concentrate ^1,10^**	288			**0.870**					**0.259**
>2 kg	221	0.04	1.04	0.870	0.27	1.31	0.82	2.08	0.257
<2 kg	67	.	.	.	.	.	.	.	.
**Body condition score ^1,11^**	763			**<0.001**					0.625
Underconditioned	83	−0.05	0.95	0.614	−0.03	0.97	0.80	1.18	0.768
Overconditioned	114	0.43	1.56	<0.001	0.08	1.08	0.92	1.27	0.357
Optimum	566	.	.	.	.	.	.	.	.
**Silage quality (at least one bunk with deficient quality < 1) ^1,12^**	661			**0.167**					**0.576**
Yes	304	0.17	1.18	0.166	0.15	1.16	0.70	1.94	0.576
No	357	.	.	.	.	.	.	.	.
**Access to water animal/water point ratio ^1,13^**	661			**0.443**					**0.944**
Meager (1.0–1.2)	90	0.023	1.02	0.897	0.04	1.04	0.74	1.47	0.819
Poor (>1.2)	267	−0.15	0.86	0.252	−0.02	0.98	0.76	1.28	0.895
Good (<1)	316	.	.	.	.	.	.	.	.

N = number of farms, OR = odds ratio, IRR = incidence rate ratio, 95% CI = 95% confidence interval. ^1^ Adjusted for herd size, farming type, breed, region, ^2^ adjusted for pasture access per group, manure scraper, robotic manure scraper, flooring type, floor soiling, moisture on floor surface, cows on contaminated floor, deep cubicles, ^3^ adjusted for flooring type, access to pasture and/or paddock, deep cubicles, manure scraper, robotic manure scraper (yes, no), floor soiling, bedding material with calcium oxide, average oblique rump length (in cm observed by the 5 largest dairy cows per farm), median neck rail position, mean width of cubicle, proportion of cows with percentage of cows with heavily soiled distal limb, ^4^ adjusted for bedding material with calcium oxide, pasture and pen access, ^5^ adjusted for herd size, farming type, breed, region, flooring type, ^6^ adjusted for manure scraper, flooring type, ^7^ adjusted for mean neck rail position, proportion of deep cubicles, ^8^ adjusted for flooring type, manure scraper, robotic manure scraper, floor contamination, ^9^ adjusted for ration type, frequency of feeding, VHHMP, animal/feeding place ratio, ^10^ adjusted for herd size, farming type, breed, region, average amount of concentrated feed per dose, VHHMP, ^11^ adjusted for ration type, frequency of feeding VHHMP (yes, no, not specified), animal/feeding place ratio, VHHMP, median days in milk, proportion of dairy’s with fat/protein ratio < 1, ^12^ adjusted for VHHMP, quality of corn silage, quality of grass silage, ^13^ adjusted for pasture access.

#### 3.2.2. Shortened Tails—Animal Level Risk Factors

After adjusting, only the fat/protein ratio in milk showed a measurable impact (*p* = 0.0394, OR = 2.21, 95% CI = 1.04–4.01) for shortened tails.

**Table 4:** Results of the univariable analyses and the adjusted models of potential risk factors associated with shortened tails on 38 German dairy farms—animal level.

	Univariable Analysis				Confounder-Adjusted Model				
Variable	N	Crude Estimate	OR	*p*-Value	Adjusted Estimate	OR	95%CI		*p*-Value
**Fat/protein ratio in milk ^1,2^**	6395	0.43	1.53	**0.182**	0.23	2.21	1.04	4.01	**0.039**
**Body condition score ^1^**	7305			**0.299**					**0.299**
Underconditioned	1713	0.13	−0.04	0.768	−0.04	0.96	0.75	1.23	0.766
Optimal	2835	0.12	−0.15	0.209	−0.15	0.86	0.68	1.09	0.209
Overconditioned	2757	.	.	**.**	.	.	.	.	.

N = number of animals, OR = odds ratio, 95% CI = 95% confidence interval. ^1^ Adjusted for herd size, farming type, breed region, ^2^ adjusted for ration type, frequency of feeding, animal/feeding place ratio, VHHMP, median days in milk, categorized body condition score.

## 4. Discussion

The present study, including data from 86,355 dairy cows on 765 farms across three regions in Germany, is comparable to a New Zealand study on risk factors, which included 92,348 cows across 200 farms and nine regions. The observations of the PraeRi study revealed 11,602 cows (13.44%) with deviated tails, and 955 cows (1.11%) with shortened tails [21].

### 4.1. Deviated Tails

#### 4.1.1. Cow-, Environment-, and Management-Related Risk Factors

Lame cows display behavioral patterns that differ substantially from non-lame animals, including a reduced number and duration of visits to the feed bunk, longer lying bouts, and a smaller number of steps per day. Lame cows tend to lose social status within the herd and experience increased social pressure from their herd mates [39]. Furthermore, inappropriate handling practices by farm staff, such as forcing cows to move, have been associated with tail injuries and compromised animal welfare [40]. At the farm level, lameness was not identified as a risk factor for deviated tails after adjustment. In order to account of the individual behavioral patterns of a lame cow and the different management practices, the model was additionally calculated at the animal level. This finding indicated a substantial association in loose housing. The OR for dairy cows that were not lame was 0.77. A comparable OR was ascertained in the adjusted model for lameness attributable to tethering. However, given the much smaller sample, this model is unlikely to be significant. A higher parity is also known as a risk factor for lameness in dairy cows [41]. The parity was not significant at farm level in the adjusted model for deviated tails, but indicates that a higher average age at herd level imply an elevated risk of deviated tails with an of 1.14. For the individual animal, the risk increased with age. Dairy cows in their first and second lactations were shown to have a significantly lower risk for deviated tails compared with multiparous cows in their fifth lactation. Primipara had a significantly lower OR of 0.53 compared with cows in their fifth lactation. Our findings are in accordance with observations from Olsen et al. (2023), who observed that multiparous cows had an increased risk of deviated tails when compared with primiparous cows [42]. The authors concluded that most tail alterations do not originate from the rearing period, but were acquired during lactation [42]. The fact that the farms were visited only once in the present project does not allow conclusions regarding when tail injuries were acquired, nor regarding the circumstances that led to them. An association between mastitis treatments and deviated tails was reported and explained by discomfort during the milking process by cows with mastitis that might refuse entering the milking parlor for intramammary treatments [42,43,44]. To develop a model that quantifies this relationship, data on mastitis treatments in this study were collected via a questionnaire and combined with animals from farms with AMS, as the treatment of mastitis on these farms involves a particularly high level of handling due to the different working structures than treatments in farms with a milking parlor. However, the model’s validity is questionable because the number of mastitis treatments in the questionnaire did not come from farm records but from farmers’ estimates, which may introduce bias. A comparison of work processes should also be conducted for the different parlor types. While no significant association was found for the overall model (*p* = 0.051), a significantly lower IRR of 0.82 was observed in rotary parlors compared to herringbone parlors. This may indicate differences in work processes and their association with deviated tails.

Forcing the dairy cows to enter the hoof trimming chute could lead to a higher risk of injuries [45]. Whether hoof trimming is performed by professional hoof trimmers or by farmers affects the number of cows that can be trimmed per day and the selection and separation procedures involved. Our hypothesis of an association between the person who performs the trimming (professional or farmer) and deviations of the tail was not confirmed in the adjusted model.

To determine whether grazing episodes in tie-stall housing systems affect the prevalence of deviated tails, distinct categories based on hours spent on pasture per day were evaluated. On farms with a maximum daily grazing time of 2 h, an increased risk of tail deviation was identified, with an IRR of 4.15. It has been hypothesized that this could be a prerequisite for enhancing the level of interaction between cows and staff members. On dairy farms with shorter grazing intervals, it is necessary to herd cows more frequently than on farms with longer grazing intervals in order to get them into the milking parlor, the stall, or the pasture. However, it is important to consider the model’s limitations. Only 4 of 83 farms were included in the grazing-for-up-to-2 h category, which could have distorted the results.

In a study on 17,893 dairy cows from 317 tie-stall farms in Ontario (CAN), tie-rail height was associated with an increase in the prevalence of broken (=deviated) tails. The prevalence increased (*p* < 0.001) by 1% for each 2.5 cm decrease in tie-rail height [24]. Slightly dirty udders were associated positively (*p* < 0.001) with broken tails (=deviated tails in the same study, while clean hind limbs and udders had a negative association (*p* = 0.001) with broken tails) [24]. The authors suggest that the tie-rail height causes dairy cows to take longer to stand up, which leads to staff using more force by twisting their tails, this may cause broken tails [24]. The restriction of the range of motion of the tail is a consequence of broken tails, that has been associated with soiling of the udder [24]. Neither the observed stall dimensions in the present study nor the cleanness of the limbs in the tie stalls was associated with a risk for deviated tails.

#### 4.1.2. Animal-Associated Risk Factors

Breed was a significant risk factor for deviated tails. In the North and East regions, German Holsteins are the predominant breed on dairy farms, while in the South region, Simmentals and Brown Swiss make up the vast majority. Simmental and Brown Swiss are dual-purpose cattle kept for milk and beef production. The investigation of breeds is predicated on two considerations. The investigation of breed differences is based on two key considerations. Firstly, anatomical differences between cattle breeds have been identified, including variations in the skeletal system examined through different skull morphologies [46], as well as variations in muscle fiber development among different cattle breeds [47]. These findings suggest differences in the passive and active components of the musculoskeletal system, which could affect the mechanical properties of the tail and the force needed to cause deviations. These differences in suggestions on anatomical variations between cattle breeds are further reinforced by the differing BCS of German Holstein, Brown Swiss and Simmental [48]. It is hypothesized that variations in the development of the musculoskeletal system may have an impact on the necessary force to deviate the tail. Secondly, a comparison of Brown Swiss and Holstein Friesian cattle revealed different temperaments during milking, suggesting that the force necessary to move them to the milking parlor also differs between different cattle breeds [49]. The analysis showed that the risk of tail deviation in Simmentals is significantly lower (IRR = 0.45) than in German Holsteins. The calmer behavior, as well as anatomical differences of Simmentals compared to other breeds, may cause the lower risk ratio for deviated tails. In addition to breeding, the overall body condition of the dairy cows was also considered in relation to over- or under-conditioning, but this did not yield a significant result. It rather seems that anatomical conditions or temperament influence the prevalence of deviated tails, but not the body condition.

The care ratio is a key factor in the occurrence of broken tails. An increase of 10 dairy cows per staff member in the care ratio results in a 5% increase in the baseline herd prevalence for deviated tails on the farm. Therefore, if the employee has to cope with an increasing workload [45], it is plausible that improper methods, such as tail twisting, are more likely to be used, resulting in deviated tails [39]. The calculation does not account for whether employees are also involved in rearing youngstock; in certain agricultural establishments, the rearing of juvenile animals is subcontracted to other farms. In instances where this is not the case, employees are likely to be responsible for a substantially larger number of cattle than their counterparts in agricultural settings where young animals are not reared. This bias could not be considered.

#### 4.1.3. Housing-Associated Risk Factors

In the natural lying position of a cow, the tail is pulled towards the udder and close to the body. If the dimensions of the cubicle are not adapted to the specific needs of cattle or if these exceed the dimensions intended for the cubicles, adequate positioning of limbs and the tail is not guaranteed [50]. As a result, the tail is positioned outside the cubicle, increasing its exposure to external influences, so herd mates may step on the tail, or it could be traumatized by the manure scraper. An evaluation of cubicle dimensions was conducted in loose housing and tie stalls. The present study concludes that none of the cubicle parameters had a significant influence on the risk of deviated tails. The hypothesis that sufficient cubicle dimensions reduce the risk of deviated tails cannot be confirmed either. However, when analyzing the type of cubicle, it was found that deep cubicles increased the risk of deviated tails; the optimal cubicle may ensure that the tail is not pulled towards the udder due to the outside curb of the cubicle. To better assess possible incorrect lying conditions, the skin lesions or swellings on the hock were used as an indicator of incorrect cubicle management, but no association could be established. The investigation further considered the hypothesis that the dairy cows’ location outside the cubicle, with cows lying within the path of other cows, was a contributing factor to the observed outcomes. However, the presence of narrow walking paths, which could hinder cow traffic and result in tail injuries, could not be verified as a risk factor, but in tie stalls where animal traffic is not a limiting factor for the available space, animal size was associated with a risk factor. As numerous factors that improve animal comfort today were not relevant when older barns were built, the date of the last modernization of the barn was also evaluated, but in the end, it did not affect the risk of deviated tails. None of the manure-removal systems was identified as a risk for deviated tails. Therefore, either no trauma is caused by manure removal systems or the trauma is so severe that tail amputation is necessary. For this reason, the manure removal systems were considered in the observations of shortened tails.

### 4.2. Shortened Tails

#### 4.2.1. Housing-Associated Risk Factors

The primary objective of the present study was to ascertain whether the presence of deviated tails constitutes a risk factor for shortened tails. A similar association was established by Volhøj et al. (2024) for the occurrence of band-shaped lesions in broken tails [51]. The analysis revealed that shortened tails were the sole risk factor to emerge with a highly significant *p*-value. The cow’s tail is well supplied with blood and innervated; the dorsal part of the tail is innervated by the plexus caudalis dorsalis, which is responsible for regulating the lifting of the tail and for the innervation of the dorsal surface of the tail skin. The nerve fibers of the plexus caudalis ventralis are responsible for regulating the drawing in of the tail and innervation of the ventral part of the tail skin; the nerve fibers are thicker and lie close to the vertebral body [52]. The available data suggest that (reduced sensitivity and mobility) ascending inflammation or restricted blood supply due to swelling can potentially lead to amputation of the tail [7,8]. Therefore, all identified risk factors for deviated tails were included in the analyses for tail amputations.

Investigations on lameness as risk factor were only possible at farm level, as the sample size at animal level was insufficient to yield robust results. As with deviated tails, the studies were unable to demonstrate any association at the farm level.

To gain an overview of the significance of the different housing systems, they were analyzed. Since a significant effect was observed for deviated tails, the influence of deep-bedded cubicles was also investigated. Overall, the model showed no effect, but the estimates were negative for straw-based loose housing and for farms exclusively equipped with deep-bedded cubicles. The housing system, therefore, appears to exert an effect on the occurrence of shortened tails. The inclusion of a manure scraper in the investigation of a shortened tail was performed, with the aim of determining whether the trauma was severe enough to necessitate amputation. For manure removal systems, various studies were carried out with regard to claw health, and also studies on the stress load on the dairies [53,54,55]. Possible injury risks were not investigated. To investigate other possible housing-associated causes of severe trauma, the following data were analyzed: The deep-bedded cubicles, the proportion of cows lying outside the cubicles, the animal-to-cubicle ratio in the herd and the parlor type. However, a significant correlation with shortened tails could not be established; only farms with consistently deep-bedded cubicles had a reduced risk (IRR = 0.7) of shortened tails, in contrast to their association with deviated tails. Consequently, the hypothesis that multiple causes for severe trauma resulting in an amputation are caused by injuries sustained from other dairy cows stepping on the tail appears to be unfounded at the farm level.

The presence of moisture and manure on the walkways of dairy cows has been demonstrated to result in the softening of claws and soiling of the skin of the distal hind limbs [56,57]. In addition, fecal soiling can lead to encrustations as faecoliths on the tail, which may form a solid circular ring around it, reducing blood flow and exerting pressure on the tail’s soft tissues due to edema [58]. Moisture and soiling of the walking paths were assessed, but no association was found. A single farm visit may not provide a comprehensive evaluation of barn cleanliness. To address this limitation, an assessment of the animals’ cleanliness was also conducted. The cleanliness of the tail was not observed in the present study, but in order to draw conclusions about the degree of soiling of the cows, the soiling of the distal phalanx was included in the study [59]. In the adjusted model, no association was demonstrated.

#### 4.2.2. Management- and Animal-Associated Risk Factors

The care ratio was identified as a significant risk factor for deviated tails in the present study. In the present study, the care ratio was not found to be a risk factor for shortened tails. It therefore appears that there are various management factors associated with the observed tail injuries. Tail tip necrosis in fattening bulls was identified when excessive amounts of starch-rich feed or substandard feed quality were offered, and in cases of high stocking densities [7,11]. The maximum concentration of concentrated feed influences the rumen microbiome; intermittent reductions in pH can lead to the death of mainly gram-negative bacteria and thus to the release of endotoxins [60]. These factors were included in the study, and the role of veterinary herd care was investigated, but no association was identified. The different effects of feeding can be seen in the fat-protein quotient for dairy cows [61]. At the farm level, significance could only be demonstrated at the univariable model; as the data was sufficient, a model could be created at the animal level. Contrary to the hypothesis that acidic metabolic states increase the risk of shortened tails, it has been demonstrated that the higher the fat-protein quotient, the higher the risk of shortened tails in a herd. The increase in the fat-protein quotient indicates a ketotic metabolic state [62]. The presence of a ketotic metabolic state within a dairy cow population is a well-documented risk factor for the development of secondary diseases [63]. This could provide a possible explanation, but it cannot be analyzed from the dataset of the present study. To a certain extent, the presence of ketone bodies in dairy cows and an increase during the transition period can be considered normal [64]. However, an excessive presence of ketonic acids is triggered by an excessive inflammatory response. The latter can alter the blood vessels of the tail, leading to reduced blood flow. Ketotic metabolic states were associated with an increased BCS at calving [65]. The analysis in this study included cows of all lactation stages to map all metabolic changes with the BCS that occur during the lactation period, but it can only show an increased BCS as a significant risk factor. As several breeds with different milk yield levels were examined in the study, which have different levels of metabolic changes associated with increasing milk yield during lactation and transition period [66], the distinct breeds were also examined. Shortened tails and breed cannot be associated, but German Holstein exhibits an elevated risk compared with other breeds.

Besides feeding, water intake is a critical aspect of dairy husbandry, which is influenced by numerous factors [67]. A lack of water intake can lead to reduced perfusion of peripheral tissues. As water intake could not be controlled in this study, the animal-to-drinking-place ratio was used as an indicator. An association could not be established.

### 4.3. Limitations

The present study’s population represented heterogeneous dairy cow farming practices in Germany and yielded reliable results by including a large number of farms. Farms were selected at random, and those selected were invited to participate in the study. Selection bias may have occurred, as some farms did not respond to the invitation to participate in the study.

On-farm evaluations were conducted during a single farm visit, precluding the possibility of tracing alterations that may have occurred over time. Due to the cross-sectional design of this study, it is not possible to establish causal relationships as temporal precedence cannot be determined. Nevertheless, the observed associations may indicate potential causal pathways and provide valuable hypotheses for further longitudinal research.

## 5. Conclusions

The results demonstrate that tail deviations are prevalent among dairy cows in Germany, independent of the region, and were associated with lameness, parity, breed, and management factors, including employee workload. Conversely, shortened tails are less common and can be interpreted as a consequence of acute tail injuries or of metabolic disorders. International farm animal welfare protocols should include tail scoring by standard procedures described. Tail assessments in the course of time under defined conditions should be performed to increase our understanding of the causes and pathogenesis of tail disorders.

## Data Availability

The data sets presented in this article are not readily available because the data were acquired through cooperation between different universities. Therefore, any data transfer to interested persons is not allowed without an additional formal contract. Data are available for qualified researchers who sign a contract with the project consortium. This contract will include guarantees of the obligation to maintain data confidentiality in accordance with the provisions of German data protection law. Currently, there exists no data access committee nor any other body that can be contacted for the data; a committee will be founded for this purpose. This future committee will consist of the authors as well as members of the related universities. Interested cooperative partners, who can sign a contract as described above, may contact R.M., Freie Universität Berlin, Institute of Veterinary Epidemiology and Biostatistics, 14163 Berlin, Germany, E-mail: roswitha.merle@fu-berlin.de. Requests to access the data sets should be directed to roswitha.merle@fu-berlin.de.
